# Advancing CAR-based immunotherapies in solid tumors: CAR- macrophages and neutrophils

**DOI:** 10.3389/fimmu.2023.1291619

**Published:** 2023-11-28

**Authors:** Yanling Liang, Qumiao Xu, Qianqian Gao

**Affiliations:** ^1^ Institute of Cancer Research, Shenzhen Bay Laboratory, Shenzhen, China; ^2^ Human Phenome Institute, Zhangjiang Fudan International Innovation Center, Fudan University, Shanghai, China; ^3^ BGI Research, Hangzhou, China; ^4^ BGI Research, Shenzhen, China

**Keywords:** CAR macrophages, CAR neutrophils, solid tumor, cancer immunotherapy, adoptive cell therapy

## Abstract

Macrophages and neutrophils are the main components of the innate immune system and play important roles in promoting angiogenesis, extracellular matrix remodeling, cancer cell proliferation, and metastasis in the tumor microenvironment (TME). They can also be harnessed to mediate cytotoxic tumor killing effects and orchestrate effective anti-tumor immune responses with proper stimulation and modification. Therefore, macrophages and neutrophils have strong potential in cancer immunotherapy. In this review, we briefly outlined the applications of macrophages or neutrophils in adoptive cell therapies, and focused on chimeric antigen receptor (CAR)-engineered macrophages (CAR-Ms) and neutrophils (CAR-Ns). We summarized the construction strategies, the preclinical and clinical studies of CAR-Ms and CAR-Ns. In the end, we briefly discussed the limitations and challenges of CAR-Ms and CAR-Ns, as well as future research directions to extend their applications in treating solid tumors.

## 1 Introduction

Chimeric antigen receptor (CAR)-engineered T (CAR-T) cell therapy has demonstrated remarkable success in the treatment of hematological malignancies (1). Since the launch of the first CAR-T product Kymriah in 2017 (2), additional five CAR-T products have been approved by the Food and Drug Administration for treating B-cell lymphomas (3). The overall response rates of CAR-T cell therapy in B-cell malignancies reached 30-70%, with some case over 90% (4). Great efforts are being made to increase the efficacies of CAR-T therapy for solid tumors, which remain challenging due to many factors, such as a lack of tumor-specific surface antigens, antigen escape, insufficient infiltration of CAR-T cells into the tumor bed, dysfunctional phenotypes in the immunosuppressive microenvironment, and poor persistence (5, 6). In addition, the acute inflammatory responses induced by CAR-T treatment raised safety concerns about the risk of severe side effects, such as cytokine release syndrome (CRS) and neurotoxicity, which in some cases were lethal (7). Thus, there is a great need to develop novel therapeutical tools to extend the benefit of CAR-based immunotherapies.

Innate immune cells play important roles in responding to pathological conditions and maintaining immune homeostasis. Among them, macrophages and neutrophils are plastic immune cells with diverse functional phenotypes responsive to different environmental signals (8). Macrophages respond to specific stimuli in the tumor microenvironment (TME) and reversibly transform into anti-tumor M1 (pro-inflammatory, classically activated macrophages) or tumor-promoting M2 (anti-inflammatory, alternatively activated macrophages) phenotypes (9–11). Likewise, neutrophils in the TME have the tendency to polarize to N1 or N2 phenotypes, which can exert anti-tumor or pro-tumor functions, respectively (12). Research on targeting or modulating macrophages and neutrophils to combat tumors has attracted much attention in the field of tumor immunology (13, 14). In this review, we mainly summarized the current studies on adoptive cell therapies using macrophages or neutrophils, the advantages, and designs of CAR-engineered macrophages (CAR-Ms) and neutrophils (CAR-Ns), their preclinical and clinical progress, limitations, and the emerging research directions for the broader applications of CAR-Ms and CAR-Ns in solid tumors.

## 2 Adoptive cell therapies using macrophages or neutrophils

### 2.1 Adoptive cell therapies using macrophages

Given the key roles of macrophages in tissue repair and inflammation, macrophage-based cell therapies have been applied in various diseases. Danon et al. treated human ulcers using macrophages prepared from human peripheral blood monocytes (15). The use of activated human macrophages for the treatment of anal fissures is currently in phase 3 trials (NCT00507364). Moroni F et al. conducted the first phase I dose-increasing trial of human autologous macrophage therapy (ISRCTN 10368050) on adult patients with liver cirrhosis. All participants survived and did not undergo transplantation for one year, providing a macrophage-based treatment strategy for liver cirrhosis and other fibrotic diseases (16).

Tumor-associated macrophages (TAMs) are the most abundant immune cells in the TME with heterogenous populations, which mostly adopted M2-like phenotypes with immune-suppressive properties (17). Therefore, for adoptive cell therapies to benefit from the intrinsic superior tumor-homing and infiltrating capabilities of macrophages, effective strategies are needed to activate macrophages properly and maintain their anti-tumor characteristics in the TME. In 1988, Dumont et al. induced macrophages from human peripheral blood monocytes and used immunostimulatory compounds to activate macrophages, which showed cytotoxicity to human tumor cell lines and ovarian tumors growing on nude mice (18). The first clinical trial of adoptive immunotherapy using autologous monocyte-induced macrophages was reported in 1990 (19). The macrophage therapy was well tolerated, and some patients experienced stable disease within 6 months after the treatment, but the primary tumor did not subside (19). The reasons that macrophages failed to eliminate the tumor may be the influence of various immunosuppressive factors in the TME (20). Thus, when unmodified macrophages are transported back to the tumor site, they may be transformed into M2-like phenotypes and lead to tumor progression (21). To address these issues, De Palma et al. genetically engineered monocytes to secrete inflammatory cytokine IFN-α. Due to their high migration and tumor-homing ability, the modified monocytes aggregated at the tumor sites and induced anti-tumor immune responses within the TME (22). Macrophages engineered to express cytokines or antibodies, including IL-12 (23, 24) and bispecific T-cell engagers (25), induced strong pro-inflammatory immune responses in the TME as well. Moreover, those engineered cells may reduce the immunotoxic effects of systemic administration of cytokines or antibodies (24).

With the advantages of high biocompatibility and phagocytic ability, studies have manipulated macrophages as drug carriers, which delivered targeted therapies to diseased tissues with improved drug stability (26, 27). In addition, compared to other types of macrophages, M1-like macrophages have stronger phagocytic ability to absorb sufficient drug-loaded nanoparticles (28), thereby enhancing the suppression of tumor growth (29). Through incubating the chemotherapy drug doxorubicin (DOX) into a mouse macrophage-like cell line RAW264.7 (30), Fu et al. showed that DOX-loaded macrophages displayed stronger anti-tumor effects than DOX alone, and suppressed the lung metastasis of mouse 4T1 breast cancer (30). To delay the toxicity of DOX to primary macrophages, Pang et al. loaded M1 macrophages derived from mouse bone marrow with nanoparticles encapsulating DOX. They showed that macrophages loaded with nanoparticles were able to infiltrate effectively through the endothelial barrier into the brain tumor tissue and exhibit significant anti-glioma effects (31). However, these macrophage-based immunotherapies described above lacked the specificity to tumor.

**Figure 1:**
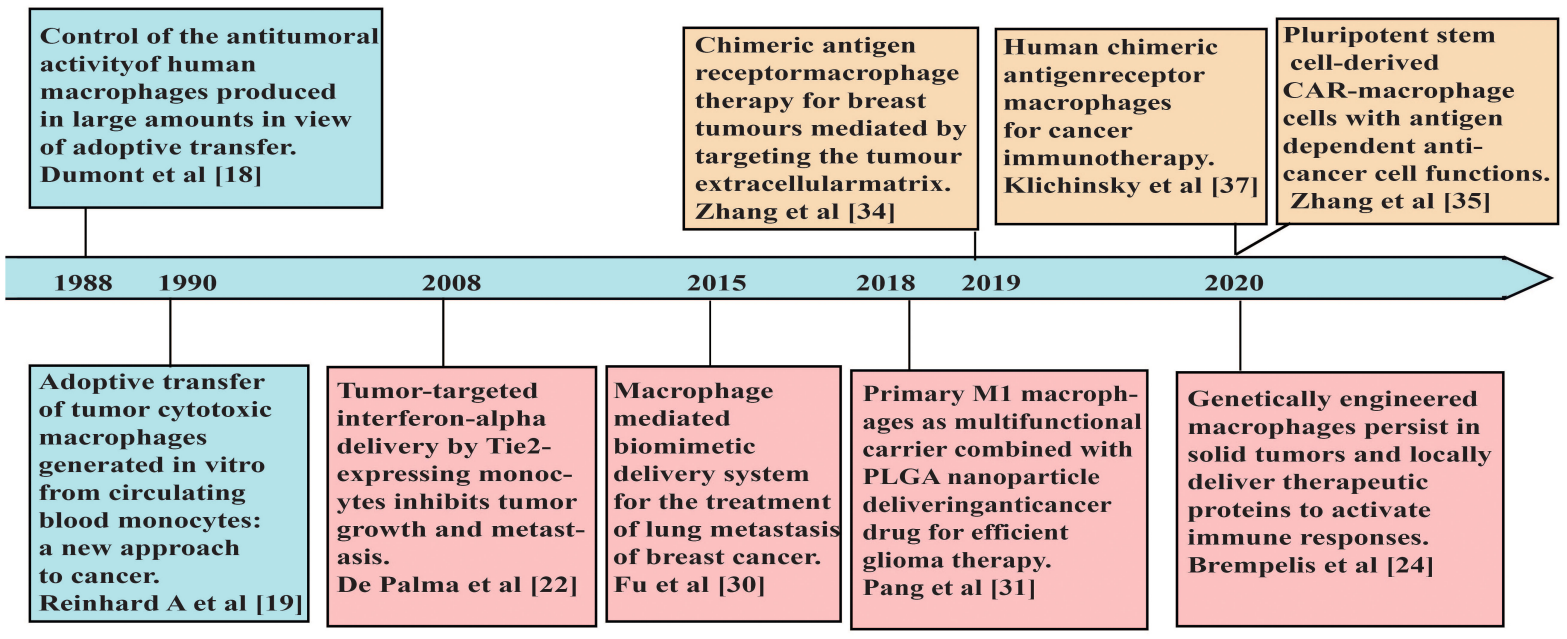
A brief timeline of representative studies using macrophages in adoptive cell therapies for cancers.

Recently, combining the unique properties of macrophages with tumor antigen-induced activation, CAR-Ms have evolved as promising adoptive cell therapies towards solid tumors (**Figure 1**) (**Table 1**). CAR-Ms maintained the pro-inflammatory phenotypes of macrophages in the TME with potent anti-tumor effects in pre-clinical studies and clinical trials (35, 37, 38, 42, 52).

**Table 1:** Research studies on CAR-Ms.

Target	Activation domain	Gene transfer method	Cell source	*In vitro* validation	*In vivo* model	Reference
CEA	Fc-γ-receptor (CD64)	Adenovirus	Human peripheral blood CD14^+^ monocytes	Human gastric cancer cell line MKN45K	/	Biglari et al., 2006 (32)
CD19; CD22	Megf10	Lentivirus	J774A.1 cells	Human lymphoblast-like cell line Raji	/	Morrissey et al., 2018 (33)
HER2	CD147	Ad5/F35 adenovirus	Raw264.7 cells	4T1-HER2	Syngeneic model of BALB/c mice via orthotopic transplantation of 4T1-HER2 cells	Zhang et al., 2019 (34)
Mesothelin; CD19	FcγRI	Lentivirus	iPSCs induced from PBMCs	Human leukemia cell line Nalm6; human ovarian cancer cell line HO8910	IP injection of cancer cells in immunodeficient NSG mice	Zhang et al., 2020 (35)
HER2	CD3ζ	Ad5/F35 adenovirus	Murine bone marrow cells	CT26-HER2 (murine colorectal carcinoma cell line CT26 overexpressing HER2)	Syngeneic model of BALB/c mice via graft of CT26-HER2 cells	Pierini et al., 2020 (36)
HER2	CD3ζ	Ad5/F35 adenovirus	Human peripheral blood CD14^+^ monocytes	Human ovarian cancer cell line SKOV3	Lung metastasis model via IV infusion of SKOV3; primary tumor model via IP injection of SKOV3 in immunodeficient NSGS mice	Klichinsky et al., 2020 (37)
CCR7	MerTK	Lentivirus	Murine bone marrow cells	Murine breast cancer cell line 4T1	Syngeneic model of BALB/c mice via subcutaneous graft of 4T1 cells	Niu et al.,2021 (38)
ALK	CD3ζ	Nanocarrier	Primary macrophages *in vivo*	Neuro-2a cells	Neuro-2a tumor-bearing mice	Kang et al.,2021 (39)
SARS-CoV-2 S glycoprotein	MERTK	Lentivirus	human monocyte line THP-1	293 cells; Vero E6 cells	/	Fu et al., 2021 (40)
CEA	CD3ζ	Lentivirus	Human Cord blood-derived HSPCs	Human fibrosarcoma cell line HT1080	/	Paasch et al., 2022 (41)
CD133	CD3ζ	Nanoparticles in hydrogel	Primary macrophages *in vivo*	Murine glioma cell line GL261; patient -derived GBM (PDG) cells	Syngeneic model of C57BL/6J mice via orthotopic transplantation of GL261 cells; Intracranial injection of PDG cells in humanized NOG-EXL mice.	Chen et al., 2022 (42)
CD19	FcRγ	Lentivirus	Murine bone marrow cells	Human lymphoblast-like cell line Raji	/	Liu et al., 2022 (43)
GD2	CD3ζ	CRISPR-Cas9	H9 hPSCs	CHLA-20 neuroblastoma cells; WM266-4 melanoma cells	CHLA-20 xenograft mouse model	Zhang et al., 2023 (44)
HER2	FcγRI	Lentivirus	Bone marrow-derived macrophages	The cell lines MC38, B16F10, ID8	The intraperitoneal tumor-bearing model; the subcutaneous tumor model; the lung metastasis model	Huo et al., 2023 (45)
HER2(ErbB2)	CD3ζ	DNA nanocarrier	Primary macrophages *in vivo*	Murine glioma cell line GL261-H	An orthotopic BSG PDX model was established by intracranial injection of patient-derived brainstem glioma cells into huHSC-NOG-EXL mice	Gao et al., 2023 (46)
SasA	CD3ζ	Peptide nanoparticle (PNP)	The locoregional *in situ* induction at the bone-implant interface	MRSA bacteria	the hematogenous orthopedic infection mouse models	Li et al.,2023 (47)
HER2; CD47	CD3ζ	Adenovirus	human monocyte line THP-1	Human ovarian cancer cell lines (SKOV3 and A2780)	Xenograft tumor model in nude mice; Syngeneic tumor-bearing NCG mice	Chen et al., 2023 (48)
HER2	FcϵR1γ	Lentivirus	Human primary peritoneal macrophages	MKN45 cells	MKN45 gastric cancer mouse peritoneal carcinomatosis models	Dong et al., 2023 (49)
GPC3	CD3ζ	Lipid nanoparticle-mediated dual mRNA co-delivery	*In situ* infection of HCC associated macrophages	Hepa 1–6 cells	The orthotopic HCC mouse model was established by inoculating Hepa1–6 cells	Yang et al., 2023 (50)
MSLN	CD3ζ	Lentivirus	iPSCs induced from PBMCs	HO-8910 ovarian cancer cells	IP injection of HO-8910 cancer cells in immunodeficient NSG mice; ovarian cancer orthotopic injection mouse model	Wang et al., 2023 (51)

CEA, carcinoembryonic antigen; CCR7, C-C motif chemokine receptor 7; FcγRI, Fcγ receptor I or CD64; MerTK, MER proto-oncogene tyrosine kinase; Megf10, Multiple EGF like domains 10; PBMCs, peripheral blood mononuclear cells; iPSCs, induced pluripotent stem cells; HSPCs, hematopoietic stem and progenitor cells; GBM, glioblastoma; IV, intravenous; IP, intraperitoneal; GD2, disialoganglioside; SasA, saureus surface protein A; ALK, anaplastic lymphoma kinase; GPC3, glypican-3, a glycosylphosphatidylinositol-anchored glycoprotein attached to the cell membrane; MSLN, mesothelin.

### 2.2 Adoptive cell therapies using neutrophils

As the most abundant immune cells in circulation, neutrophils play central roles in the early immune responses to tissue damage or infection (53). Granulocyte infusion for the treatment of refractory neutropenia sepsis is an approved neutrophil therapy (54).

Activated neutrophils secreted various pro-inflammatory cytokines and activated T cells by antigen presentation (55). They also presented high mobility and ability to infiltrate tissues that were hard for other cells to enter, such as the blood-brain barrier (BBB) (56–58), and could be used as effective drug carriers. Xue et al. reported that neutrophils isolated from the mouse bone marrow carrying paclitaxel liposomes could penetrate the BBB and inhibit glioma recurrence in glioblastoma mouse models (59).

However, under the effects of tumor suppressive microenvironment and cytokines, neutrophils in the TME are mostly polarized to acquire N2-like phenotypes, with tumor-promoting functions through driving angiogenesis, extracellular matrix remodeling, metastasis, and immune suppression (60). Thus, cell therapy strategies using neutrophils need to consider regulating the intrinsic properties of neutrophils to effectively trigger anti-tumor responses (61). Leslie et al. suggested that infusion of immature neutrophils isolated from lipopolysaccharides (LPS)-treated mice was sufficient to reactivate the anti-tumor immunity in a hepatocellular carcinoma (HCC) mouse model (62). Akin to CAR-Ms, recent studies applied CAR engineering in neutrophils, which activated the anti-tumor functions of neutrophils, maintained the N1-like phenotypes, and drove strong proinflammatory immune responses in the TME (57, 58, 63). These studies have demonstrated promising efficacies of CAR-Ns in mouse models with glioblastoma (57, 58, 63). The exciting advance in CAR-Ns may expand the toolbox of CAR-based immunotherapies and improve the outcomes of cancer treatment.

## 3 Construction of CAR-Ms and CAR-Ns

### 3.1 CAR design

**Figure 2:**
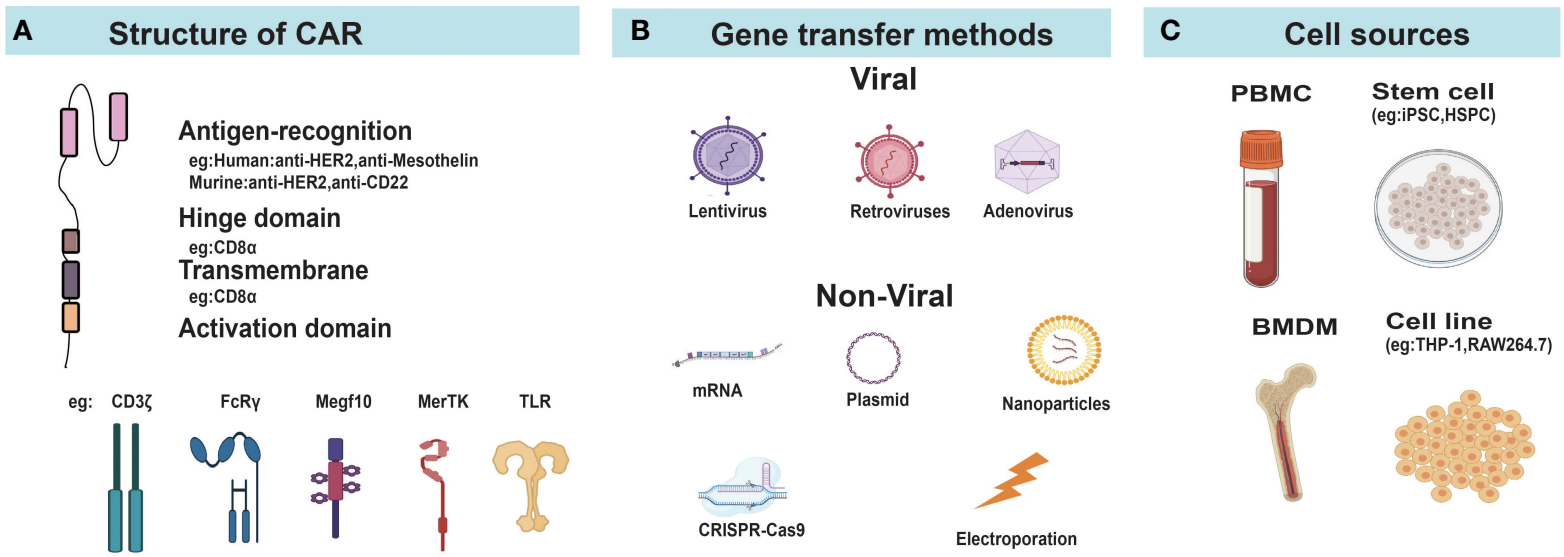
Construction of CAR-Ms and CAR-Ns. **(A)** Diagram of CAR structure. **(B)** Different gene transfer methods for delivering CAR genes into cells. **(C)** The main cellular sources for CAR-Ms and CAR-Ns. FcRγ, Fcγ receptor; MerTK, MER proto-oncogene tyrosine kinase; Megf10, Multiple EGF like domains 10; TLR, Toll-like receptors; PBMC, peripheral blood mononuclear cells; iPSC, induced pluripotent stem cells; HSPC, hematopoietic stem and progenitor cells; MSLN, mesothelin; BMDM, bone marrow derived macrophage.

The basic principles of CAR design in the field of CAR-T cells were applicable to macrophages and neutrophils. Current studies in CAR-Ms and CAR-Ns mostly used classical CAR design consisting of an extracellular antigen recognition domain, a hinge domain, a transmembrane domain, and one or more cytoplasmic signal domains (**Figure 2A**). CAR-Ms or CAR-Ns were directed to well-established tumor-associated antigens, such as CD19 (64), HER2 (65), and PSMA (63) through single chain variable fragments of cognate antibodies. The intracellular activation domain from CD3ζ was also used in CAR-Ms and CAR-Ns, as the phosphorylated sites of immunoreceptor tyrosine-based activation motif (ITAM) in CD3ζ can recruit the Syk kinase which activates downstream phagocytosis and proinflammatory signaling pathways, similar to the function of Zap70 in T cells (33, 66). To further improve the phagocytosis of CAR-Ms, Liu et al. incorporated the intracellular signaling domains from FcRγ (Fc receptor γ), Megf10 (multiple EGF-like domains protein), and PI3K (phosphoinositide 3-kinases) respectively in CAR, and showed that CAR-Ms with the FcRγ intracellular domain were more powerful in phagocytizing and killing cancer cells (43). In another study, Morrissey et al. tested the signaling domains from five known mouse phagocytic receptors, including Megf10, FcRγ, Bai1 (adhesion brain-specific angiogenesis inhibitor-1), MerTK (tyrosine protein kinase Mer), and CD3ζ in CAR-Ms. Consistently, CAR-Ms with the signaling domain of FcRγ were most effective in phagocytosis, which was further promoted by adding a tandem PI3K recruitment domain (33). Similar studies in CAR-Ns need to be conducted to optimize the CAR design and enhance their functions.

### 3.2 Gene transfer strategies

Due to the high expression of pattern recognition receptors and non-proliferative nature of macrophages and neutrophils, they are more resistant to gene transfer compared to T cells (57, 67, 68). Different strategies based on virus, electroporation, and nanoparticles have been developed for the delivery of CAR genes to macrophages or neutrophils (**Figure 2B**).

#### 3.2.1 Virus-mediated gene transfer

Lentivirus and retrovirus vectors can mediate the integration of transgenes into host genomes with high efficiencies and are commonly used for stable gene transfer to T cells. Though the primary mouse bone marrow derived macrophages (BMDMs) were infected by human immunodeficiency virus type 1 (HIV-1) lentivirus at high efficiency (64, 69), studies reported that transduction of human primary myelocytes were impeded by bone marrow specific restriction factor SAMHD1 (SAM domain and HD domain-containing protein 1) (69, 70). The virion-associated protein Vpx from HIV-2 or related simian immunodeficiency viruses was found to induce SAMHD1 degradation, which increased the infection efficacy of lentivirus to macrophages (71). Later studies used unmodified lentivirus to transduce human induced pluripotent stem cells (iPSCs) or CD34^+^ hematopoietic stem cells, which were then differentiated into macrophages (See section 2.3). Retroviruses (excluding the lentivirus subclass) are generally considered ineffective in transfecting non-proliferative or poorly proliferating cells (72), and the infection efficiency of wild-type retroviruses to macrophages was low (73). Introducing the nuclear localization signal sequence into the matrix protein of the C-type retrovirus spleen necrosis virus greatly increased the proportion of macrophages infected by the modified retrovirus (74). Roberts et al. reported that the retroviral vector rkat43.2, a modified variant of the retroviral transduction system rkat4 derived from moloney murine leukemia virus transduced hematopoietic stem cells to express chimeric immune receptors with over 50% efficiency. These cells were then differentiated into neutrophils with antigen-specific cytotoxicity (75). Although current studies on CAR-Ms and CAR-Ns have not utilized such modified lentivirus or retroviruses described above, they provided useful tools for future applications.

Adenovirus (AdV) belongs to the family of double-stranded DNA viruses (76). AdV vectors have the abilities to infect both dividing and nondividing cells, and are free from the risk of genomic mutations resulting from integration into the host genome (77). Gene transfer to macrophages through the common AdV serotype 5 was challenging, and the replication-incompetent chimeric AdV vector (Ad5/F35) which incorporates the knob and shaft of adenovirus serotype 35 was constructed and able to effectively infect macrophages through interacting with the cell surface protein CD46 (78, 79). In addition, Ad5/F35 activated macrophage inflammasome and provided beneficial pro-inflammatory signals, promoting the M1 polarization of CAR-Ms (37).

#### 3.2.2 Nonviral gene transfer

With the rapid development of gene editing technologies, site-directed knock-in of CAR gene via CRISPR/Cas9 system provided a convenient strategy to perform genetic modifications (80). Zhang et al. inserted anti-GD2 CAR into the safe harbor *AAVS1* gene locus of human pluripotent stem cells (hPSCs) by CRISPR/Cas9 system through electroporation followed by differentiation into macrophages with M1-like phenotypes (44). Similar strategies were applied in CAR-Ns (57, 58, 63). Chang et al. inserted the CAR construct into the *AAVS1* site through CRISPR/Cas9 in human pluripotent stem cells and achieved stable and universal expression on differentiated neutrophils (57). Moreover, no insertion or deletion mutations were detected in predicted off-target sites (57). These studies suggested the applicability of CRISPR/Cas9 gene editing in generating CAR-Ms or CAR-Ns. New strategies using nanoparticles are discussed in the Section 6.

Generally, virus-mediated gene transfer strategies result in high transduction efficiency, but are limited by the sizes of exogenous genes that could be packaged and delivered (approximately 7.5 Kb, 8 Kb, and 6.5 Kb for lentivirus, retrovirus, and adenovirus, respectively). Non-viral methods can deliver larger fragments of transgenes with flexibility, and lower the manufactural time and costs (81). The properties of different gene-transfer methods are compared in **Table 2**.

**Table 2:** Different gene transfer methods.

	Lentivirus	Ad5/F35 adenovirus	CRISPR-Cas9- electroporation	Nanoparticles
Advantage	Well-known system, mediate long-term gene expression, infect both dividing and non-dividing cells	High transduction efficiency, transient gene expression without inserting into host chromosomes, infect both quiescent and dividing cells	High efficiency, site-directed insertion of transgene	Can encapsulate large segments of genetic materials, multiple types of cargos (DNA, RNA, protein), can be delivered *in vivo* to reduce manufactural time
Safety	Risk of insertion mutation, elicit relatively weak immune responses	Free from the risk of genomic mutations, can elicit strong immune response in animals	Possible off-target events	LNPs can potentially induce an immune response due to their foreign nature and the presence of certain lipid components
Disadvantage	Limited gene insertion size and complex virus packaging technology, prolonged expression leads to risk of on tumor, off-target events	Limited gene insertion size, technically demanding and time-consuming on viral production	Special equipment, damage to cells and high cell mortality rate from electroporation	Transfection efficacy or level of expression is substantially lower than viral vectors
Reference	(33) (35) (38) (40) (41) (43) (49) (51)	(32) (34) (36) (37) (48)	(57) (58) (63) (44)	(39) (42) (46) (47) (50)

### 3.3 Cellular sources

Human monocytic cell line THP-1 could be induced to M1-like macrophages when cultured in the presence of phorbol myristate acetate (PMA) followed by resting and treatment by LPS and IFN-γ (82), providing a convenient resource to generate CAR-Ms for *in-vitro* and animal studies. Since primary macrophages in blood is scarce, peripheral blood monocytes represent the main resource for CAR-Ms used in pre-clinical and clinical studies (**Figure 2C**). Klichinsky et al. streamlined the process of generating CAR-Ms from peripheral blood monocytes, which differentiated CD14^+^ monocytes to M1-like macrophages in the presence of GM-CSF, and then transduced them by Ad5/F35 AdV vectors (37). However, it required a large quantity of monocytes for clinical treatment, raising some concerns about the number and function of circulating monocytes in cancer patients which may be affected or impaired by previous treatments, especially in advanced cancer patients (83, 84).

Stem cells provide alternative cell resources to produce CAR-Ms or CAR-Ns. They can be stably transduced to express CAR genes and then differentiated into macrophages or neutrophils. Senju et al. derived macrophages from human iPSCs in 2011 and constructed the prototype of CAR-Ms expressing only the antigen-recognition extracellular domain, lacking the intracellular signaling domains (85). Zhang et al. transduced iPSCs with lentivirus and obtained CAR-Ms with over 50-fold expansion using differentiation factors (bFGF, M-CSF, GM-CSF, etc.), which showed significant anti-cancer effects in mouse xenograft tumor models (35). Paasch et al. transduced human cord blood CD34^+^ hematopoietic stem and progenitor cells (HSPCs) using lentivirus to generate CAR-HSPCs, which were then differentiated into functional CAR-Ms in the presence of M-CSF/GM-CSF/IL-3 (41). Roberts et al. in 1998 first reported the generation of neutrophils with cytolysis activities from hematopoietic stem cells (75). Recently, a new chemically defined, feeder-free platform was developed, which allowed robust sequential differentiation of hematopoietic progenitor cells, myeloid progenitor cells and then CD16^+^ neutrophils from hPSCs using stage-specific signaling modulators *in vitro* (57). With the infinite self-renew capability and the potential to differentiate into diverse cell types, iPSCs represent an unlimited cell source for cell therapies (86). However, the yield and function of the final products could be largely affected by the different induction and differentiation strategies, while no widely accepted standards exist in the field of iPSCs. It is urgently needed to establish a standardized scheme to allow the production of iPSC-derived macrophages and neutrophils with high reproducibility and scalability (86).

## 4 Effector functions of CAR-Ms and CAR-Ns

**Figure 3:**
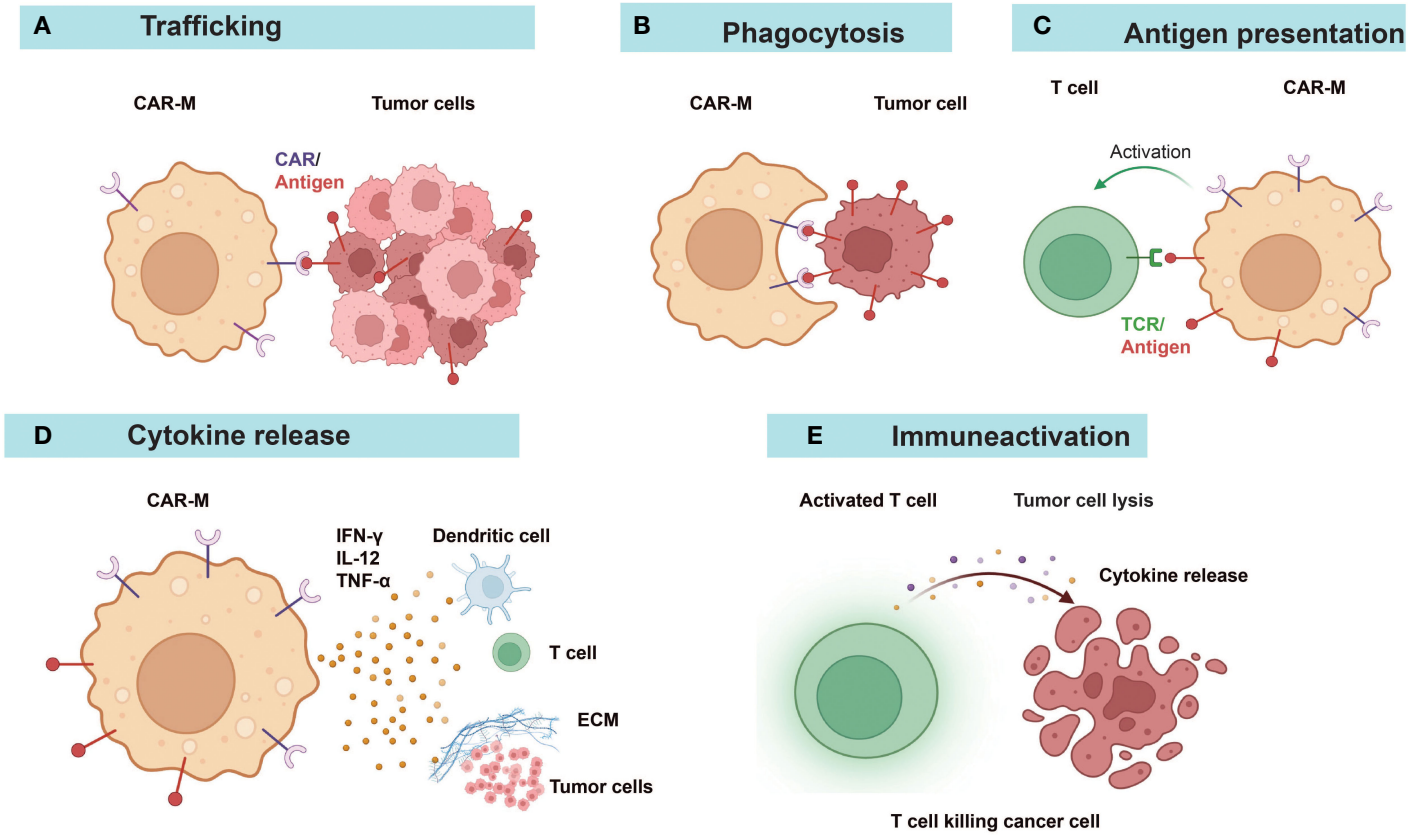
Diverse anti-tumor mechanisms of CAR-Ms. **(A)** CAR-Ms enter the TME and recognize tumor antigens. **(B)** Activation of CAR-Ms enhances the phagocytic activity of macrophages against tumors and **(C)** improves their antigen-presenting ability to T cells. **(D)** Secretion of pro-inflammatory cytokines alters the immune microenvironment. **(E)** Effector T cells activated by the functions of antigen-presentation and cytokines of CAR-Ms target and kill tumor cells. ECM, extracellular matrix.

Research studies on CAR-Ms and CAR-Ns were listed in **Table 1** and **Table 3**. Various reports have demonstrated the multifaceted anti-tumor activities of CAR-Ms (88, 89) (**Figure 3**). Upon recognizing cognate antigens on tumor cells, CAR-Ms could directly clear tumors through phagocytosis. Moreover, activated CAR-Ms could secret inflammatory factors, such as IFN-γ, IL-12, and TNF-α, to promote M1 polarization and activate antigen-presenting cells (APCs) in the TME. They also upregulated the expression of major histocompatibility complex II (MHC-II), CD68, and CD80 and CD86 co-stimulatory molecules, and served as APCs to activate T cells into effector T cells to control tumor growth. Matrix proteases produced by CAR-Ms could degrade compact tumor extracellular matrix and improve the infiltration of immune cells. Furthermore, CAR-Ms could induce the epitope spreading effect and long-term immunological memory, as shown in a study that they were able to control the growth of antigen-negative tumors in the CT26 tumor model and protect the mice from tumor rechallenge (90). In addition, nitric oxide (NO) released from IFN-activated macrophages expressing inducible NO synthase (iNOS) contributed to the inflammatory death of immune-invasive tumors (91, 92).

**Table 3:** Research studies on CAR-Ns.

Target	Activation domain	Gene modification	Cell source	*In vitro* validation	*In vivo* model	Reference
MMP2, IL13Rα2	CD3ζ	CRISPR-Cas9	hPSCs	Human GBM cell line U87MG, patient-derived GBM cells	*in situ* xenograft model via intracranial injection of luciferase-expressing GBM cells in immunodeficient NRG mice	Chang et al. (57) Chang et al. (58)
PSMA	CD3ζ	CRISPR-Cas9	hPSCs	Human prostate cancer cell line LNCaP	/	Harris et al. (63)

MMP2, matrix metallopeptidase 2, the target identified on GBM for chlorotoxin (57). Chlorotoxin is a 36 amino acid peptide found in scorpion venom and was used in CAR-T as the extracellular antigen recognition module to target GBM (87). PSMA, prostate-specific membrane antigen. hPSCs: human pluripotent stem cells.

**Figure 4:**
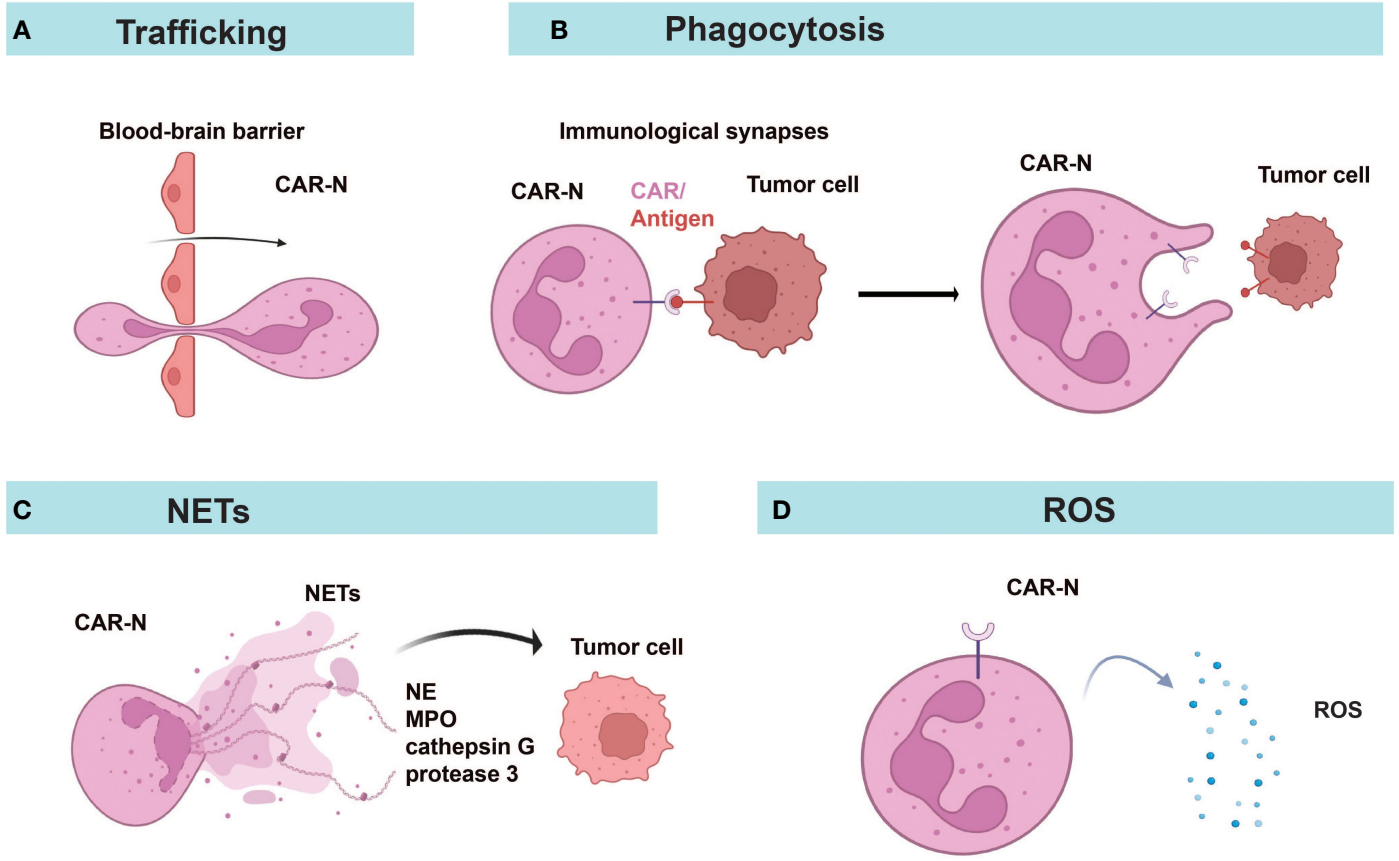
Diverse anti-tumor mechanisms of CAR-Ns. **(A)** CAR-Ns penetrate the BBB and enter the TME. **(B)** Immunological synapse formation and phagocytosis. **(C)** CAR-Ns produce NETs (neutrophil extracellular traps) and release content, such as neutrophil elastase (NE) and myeloperoxidase (MPO). **(D)** Secretion of reactive oxygen species (ROS) from activated CAR-Ns alters the immune microenvironment. NETs, neutrophil extracellular traps; NE, neutrophil elastase; MPO, myeloperoxidase; ROS, reactive oxygen species.

CAR-Ns mediated tumor lysis through phagocytosis, reactive oxygen species (ROS) production, and neutrophil extracellular traps (NETs) (**Figure 4**). Chang et al. demonstrated that CAR constructs sustained the anti-tumor N1-like phenotype, enhanced anti-tumor and tumor-infiltrating activities of neutrophils, leading to excellent efficacy in the treatment of GBM (57). Consistent with a previous report (93), only neoplastic cells were killed while healthy cells were not attacked by activated neutrophils, indicating the good safety profile of CAR-Ns (57). Upon engagement with tumor cells, immunological synapses were formed and Syk-vav1-Erk signaling pathway was activated, which induced the phagocytic activity of neutrophils and the release of ROS (57). Through comparing CAR-Ns with wild-type neutrophils, NK cells, and CAR-engineered NK cells, the study showed that CAR-Ns significantly inhibited tumor growth and prolonged the survival of mice with orthotopic GBM xenotransplant, highlighting the potential of CAR-Ns as a novel CAR-based cell therapy (57).

## 5 CAR-Ms and CAR-Ns as carriers for other therapeutic modalities

Novel strategies utilizing the tumor-homing tendency of macrophages to deliver therapeutic substances (cytokines, antibodies, chemotherapy drugs, etc.) hold the potential to further enhance the anti-tumor effects of CAR-Ms while constraining the cytotoxic effects mainly within the local tumor ecological niche and reducing the toxic effects of systemic administration (89). For example, macrophages secreting the pro-inflammatory cytokine IL-12 activated T-cell responses and prolonged survival of mice with GBM and melanoma without causing systemic toxicity (24). Macrophages releasing anti-EGFR antibodies effectively engulfed tumor cells expressing EGFR through antibody-dependent cell-mediated cytotoxicity (94). Moreover, CAR-Ms could be engineered with special control systems to release the cargo as directed. In a recent study, Liu et al. developed light-controlled CAR-Ms for drug delivery in the central nervous system (95). They showed that CAR-Ms efficiently penetrated the BBB and delivered the drug as controlled by near-infrared excitation (95).

A recent study reported that CAR-Ns carrying SiO_2_ nanoparticles loaded with the chemo-drug tirapazamine infiltrated to brain tumors and cleared the external tumor cells by phagocytosis (58). As neutrophils underwent apoptosis *in vivo*, they released the nanoparticles which resulted in effective killing of cancer cells located in the tumor core. It was estimated that 20% of nanomedicine was delivered to brain tumors through CAR-Ns, which far exceeded the conventional delivery efficiency of nanomedicine through the circulatory system (<1%) (58).

## 6 Clinical progress on CAR-Ms

Based on promising results in preclinical studies (37), Carisma Therapeutics initiated the first phase I multi-center clinical trial of CAR-Ms cell therapy (CT-0508) treating HER2-expressing solid tumors, including breast cancer, esophageal cancer, cholangiocarcinoma, ovarian cancer, and salivary carcinoma (NCT04660929). The updated clinical results in 2022 showed that 5 out of 9 participants achieved stable disease as the best overall response (96). CT-0508 showed no dose limiting toxicity, no severe CRS, or major organ toxicity. Myeloid Therapeutics initiated in September 2023 a phase I clinical trial of MT-302 targeting TROP2 through *in vivo* programming of myeloid cells using LNP encapsulating mRNA, administered to patients with advanced or metastatic epithelial tumors (NCT05969041). Another study aiming to determine the efficacy of newly developed CAR-Ms therapy using tumor organoids derived from breast cancer patients (NCT05007379) has not yet started.

## 7 Limitations and future directions

CAR-Ms and CAR-Ns possess unique characteristics that may help overcome some obstacles faced by CAR-T cell therapy, as they are expected to improve tumor infiltration, alleviate the immune-suppressive TME, and activate anti-tumor immune responses. Currently, they are still in the early stages as potential therapeutics with limited clinical data to evaluate their efficacies and safety profiles. Safety issues and various challenges need to be addressed to further expand their applications (**Table 4**). For example, activated macrophages could release large amounts of proinflammatory cytokines, such as IL-1, and IL-6, which might cause severe adverse events like CRS. Compared to the high proliferative capability of T cells, macrophages and neutrophils can hardly expand *ex vivo* or after infusion since they are terminally differentiated cells (97, 98). To obtain large quantities of autologous cells for treatment, especially from patients, might be a great challenge. They also have relatively short life cycles (99, 100), and probably lack persistency (101). Using iPSCs or HSPCs to generate enough functional CAR-Ms or CAR-Ns may be one of the strategies to address the problem with limited cell sources, and to achieve long-term tumor regression through repeated infusion. Besides, Dong et al. reported that M2-like peritoneal macrophages isolated from malignant ascites of gastric cancer patients could be transfected with CAR genes to prepare CAR-Ms, which exhibited M1-like polarization with anti-tumor and pro-inflammatory phenotypes, and promoted T cell proliferation (49).

**Table 4:** Comparison of CAR-Ms and CAR-Ns.

	CAR-Ms	CAR-Ns	
Cell source	PBMC, umbilical cord blood, bone marrow, iPSCs, THP1 cell line	hPSCs
Gene modification method	Viral (lentivirus, adenovirus) and nonviral methods (electroporation, nanoparticles)	Electroporation(CRISPR-Cas9)	
Anti-tumor effect	Phagocytosis, antigen presentation, enhance T cell cytotoxicity	Phagocytosis, ROS production and NET formation
Advantage	High infiltration to tumor, low off-tumor toxicity, low neurotoxicity	High infiltration to tumor, ability to cross the BBB, low off-tumor toxicity, no CRS, low neurotoxicity
Limitation	Short life cycle, unable to proliferate, difficulty in gene editing	Short life cycle, unable to proliferate, difficulty in gene editing, rely on *in vitro* differentiation from stem cells
Clinical trial	NCT04660929, NCT05007379, NCT05969041	/

### 7.1 *In vivo* production of CAR-based cells

To expedite the manufactural processes and reduce the cost, *in vivo* production of CAR-based therapies is under active investigations recently using nanoparticles (39, 42, 46, 50, 102). CD5-targeted lipid nanoparticles (LNPs) encapsulated with mRNAs were intravenously injected into a mouse model of heart failure, which reduced fibrosis and restored cardiac function through generating CAR-T cells targeting FAP (fibroblast activation protein) transiently *in vivo*. Similarly, Yang et al. delivered mRNAs encoding anti-GPC3 CAR and CD24-blocking protein in LNPs to liver macrophages, which elevated their phagocytic function and reduced tumor burden in an HCC mouse model (50). To improve the specificity of gene delivery to macrophages, Gao et al. incorporated the RP-182 peptide to the shell of nanoparticles, which activated phagocytosis and autophagy in M2-like TAMs via the mannose receptor CD206, and reprogrammed them into an antitumor M1-like phenotype (46). Plasmid DNA encoding ErbB2/Her2-specific CAR was delivered to macrophages by injecting the nanoparticles intratumorally, thereby generating CAR-Ms *in situ* as “living” cures which cleared the invasive tumor cells in mice with glioma (46). It is conceivable to generate CAR-Ns *in vivo* as studies have reported targeting neutrophils using nanoparticles to modify neutrophil function or deliver drugs (58, 103). *In vivo* engineering of immune cells avoided the lengthy, complex, and expensive *in vitro* production processes (50). Due to the stability and ease of manufacturing nanoparticles, it is possible to simplify long-term storage and reduce costs (46). Intratumoral administration of nanoparticles could directly target macrophages or neutrophils *in situ* and restrict the potential side effects of systemic infusion, while it is challenging in clinical applications. Experiences from intravenous injection of LNPs carrying mRNAs as vaccines might shed light on the future use of nanoparticles to generate CAR-based cells *in vivo* in humans (104). Due to the transient expression of exogenous genes, multiple doses are likely needed to produce potent therapeutic effects.

### 7.2 Functional optimization of CAR-Ms and CAR-Ns

Optimization of CAR design through assessing signaling domains and their combinations as stimulation or activation domains may further enhance the functions and reduce safety risks for CAR-Ms or CAR-Ns. For instance, various molecules are involved in functional pathways of phagocytosis, polarization, or proliferation in macrophages or neutrophils, such as the glycoprotein receptor dectin-1 (Dectin-1) (105) and MyD88 (106). The signaling domains of different receptors or signal-junction proteins are worth testing in CAR-Ms or CAR-Ns in future studies.

Various studies have implemented strategies modulating metabolic programs in T cells to enhance the functions of CAR-T cells (107). Through CRISPR screening of metabolic genes, Wang et al. found that Kelch-like ECH-associated protein 1 (KEAP1) played an important role in the pro-inflammatory activity of macrophages through inhibiting the production of itaconate. As Aconitate Decarboxylase 1 (ACOD1) is the sole enzyme to generate itaconate, ACOD1 depletion promoted pro-inflammatory activity of macrophages and enhanced the function of CAR-Ms derived from human iPSCs in solid tumors (51). Further research on the effects of metabolites is likely to discover more strategies to improve anti-tumor efficacies of CAR-Ms and CAR-Ns through metabolic reprogramming.

### 7.3 Combinatory therapies

Due to the limitations of single therapy in cancer treatment, combinatory therapies have become the long-term trend to increase the efficacies of CAR-based cell therapies. As noted above, CAR-Ms and CAR-Ns delivering drugs had combined anti-tumor effects of cell therapies and cytotoxicity from chemo-drugs (58, 95). Radiotherapy has been combined with CAR-T cells to avoid the tumor escape resulted from CAR-targeted antigen loss (108, 109). Pre-exposure to radiotherapy could induce immunogenic cell death and antigen release, promote HLA or CAR target expression on tumor cells, increase APC activation and immune cell infiltration, thereby reshaping the TME to favor anti-tumor immune responses (108, 110, 111). It is worth of evaluating radiotherapy combining with CAR-Ms or CAR-Ns in future studies.

Combination of different cell therapies can present synergistic anti-tumor effects. Mice treated with CAR-Ms and T cells together showed better anti-tumor responses compared to those treated with either cell therapy alone (37). This may be due to the enhanced anti-tumor activity of T cells induced by CAR-Ms. Further experiments showed that CAR-Ms cross-presented intracellular tumor-derived antigens after phagocytosis of tumor cells (112). Furthermore, another study demonstrated that CAR-Ms and CAR-T cells exhibited synergistic cytotoxicity against tumor cells *in vitro* (43). The inflammatory factors secreted by CAR-T cells increased the expression of co-stimulatory ligands CD86 and CD80 on CAR-Ms and enhanced the cytotoxicity of CAR-Ms by inducing M1 polarization (43).

In addition, CAR-Ms combined with antibodies, such as anti-PD-1, anti-CD47, anti-FcγRIIB and anti-Siglec-10 promoted the anti-tumor efficacies compared with CAR-Ms alone, either through enhancing the phagocytosis of macrophages or cytotoxic T cell responses (48, 113–119). Given the complexity and heterogeneity of solid tumors, it will be necessary to explore different combinatory strategies with CAR-Ms or CAR-Ns under the specific context of the TME and in clinical trials.

## 8 Conclusion

In conclusion, CAR-Ms and CAR-Ns have emerged as promising cell therapies with unique characteristics to combat solid cancers. Beyond cancers, they also provide novel treatment options for other diseases. For example, Li et al. utilized a surficial nanoparticle coating that locoregionally generated super bactericidal CAR-Ms to eradicate *Staphylococcus aureus* in mouse models, which presented a potential approach for the prevention and treatment of periprosthetic joint infection (47). Combinatory therapies with other therapeutic modalities, such as antibodies, CAR-T cells, radiotherapy, and chemotherapy are exciting directions attracting a lot of attention. Novel strategies are to be developed to further enhance the *in vivo* function and persistency of CAR-Ms and CAR-Ns, with effective manufactural routes to improve the yield of engineered cells and reduce the cost and time. More clinical studies are eagerly awaited to comprehensively evaluate the potency and toxicity of CAR-Ms and CAR-Ns in different diseases including solid cancers.

## Author contributions

YL: Writing – original draft, Writing – review and editing. QX: Writing – original draft, Writing – review and editing. QG: Writing – original draft, Writing – review and editing.
